# Evidence of local and regional freshening of Northeast Greenland coastal waters

**DOI:** 10.1038/s41598-017-10610-9

**Published:** 2017-10-13

**Authors:** Mikael K. Sejr, Colin A. Stedmon, Jørgen Bendtsen, Jakob Abermann, Thomas Juul-Pedersen, John Mortensen, Søren Rysgaard

**Affiliations:** 1 Arctic Research Centre, Ny Munkegade, Aarhus University, 8000 Aarhus C, Denmark; 2 Department of Bioscience, Vejlsøvej 25, Aarhus University, 8600 Silkeborg, Denmark; 3 National Institute of Aquatic Resources, Technical University of Denmark, Building 202, Kemitorvet, 2800 Kgs. Lyngby, Denmark; 4 ClimateLab, Fruebjergvej 3, box 98, 2100 Copenhagen O, Denmark; 5 Asiaq PO Box 1003 Qatserisut 8, 3900 Nuuk, Greenland; 6 Greenland Climate Research Centre, Greenland Institute of Natural Resources, Kivioq 2, 3900 Nuuk, Greenland; 7 Centre for Earth Observation Science, 584 Wallace Bldg, University of Manitoba, Winnipeg, MB R3T 2N2 Canada

## Abstract

The supply of freshwater to fjord systems in Greenland is increasing as a result of climate change-induced acceleration in ice sheet melt. However, insight into the marine implications of the melt water is impaired by lack of observations demonstrating the fate of freshwater along the Greenland coast and providing evaluation basis for ocean models. Here we present 13 years of summer measurements along a 120 km transect in Young Sound, Northeast Greenland and show that sub-surface coastal waters are decreasing in salinity with an average rate of 0.12 ± 0.05 per year. This is the first observational evidence of a significant freshening on decadal scale of the waters surrounding the ice sheet and comes from a region where ice sheet melt has been less significant. It implies that ice sheet dynamics in Northeast Greenland could be of key importance as freshwater is retained in southward flowing coastal currents thus reducing density of water masses influencing major deep water formation areas in the Subarctic Atlantic Ocean. Ultimately, the observed freshening could have implications for the Atlantic meridional overturning circulation.

## Introduction

The Arctic Ocean has increased its freshwater content significantly the past decade^1^ and is freshening at a rate of approximately 600 km^3^ per year^2^. A simultaneous freshening is expected in Greenland coastal waters where current rates of ice loss from the Greenland Ice Sheet have more than doubled compared to rates from 1983–2003^3^. The annual net loss of ice is estimated at 186 Gt (i.e. 186 km^3^) whereas the total ice loss during the seasonal melt in summer exceeds 1200 km^3^ annually^4^. Although much of the ice loss to date can be attributed to glaciers in West and Southeast Greenland it is now evident that there is also accelerating ice loss in Northeast Greenland^5^. In addition to contributing to global sea level rise^6^, increasing freshwater contribution from Greenland may impact local and global circulation patterns. Transport of freshwater in Greenlandic coastal currents and into the surface waters of the subarctic Atlantic could influence deep water convection and potentially the Atlantic meridional overturning circulation^7,8^. This represents an important potential climatic feedback mechanism not included in current IPCC models.

**Figure 1:**
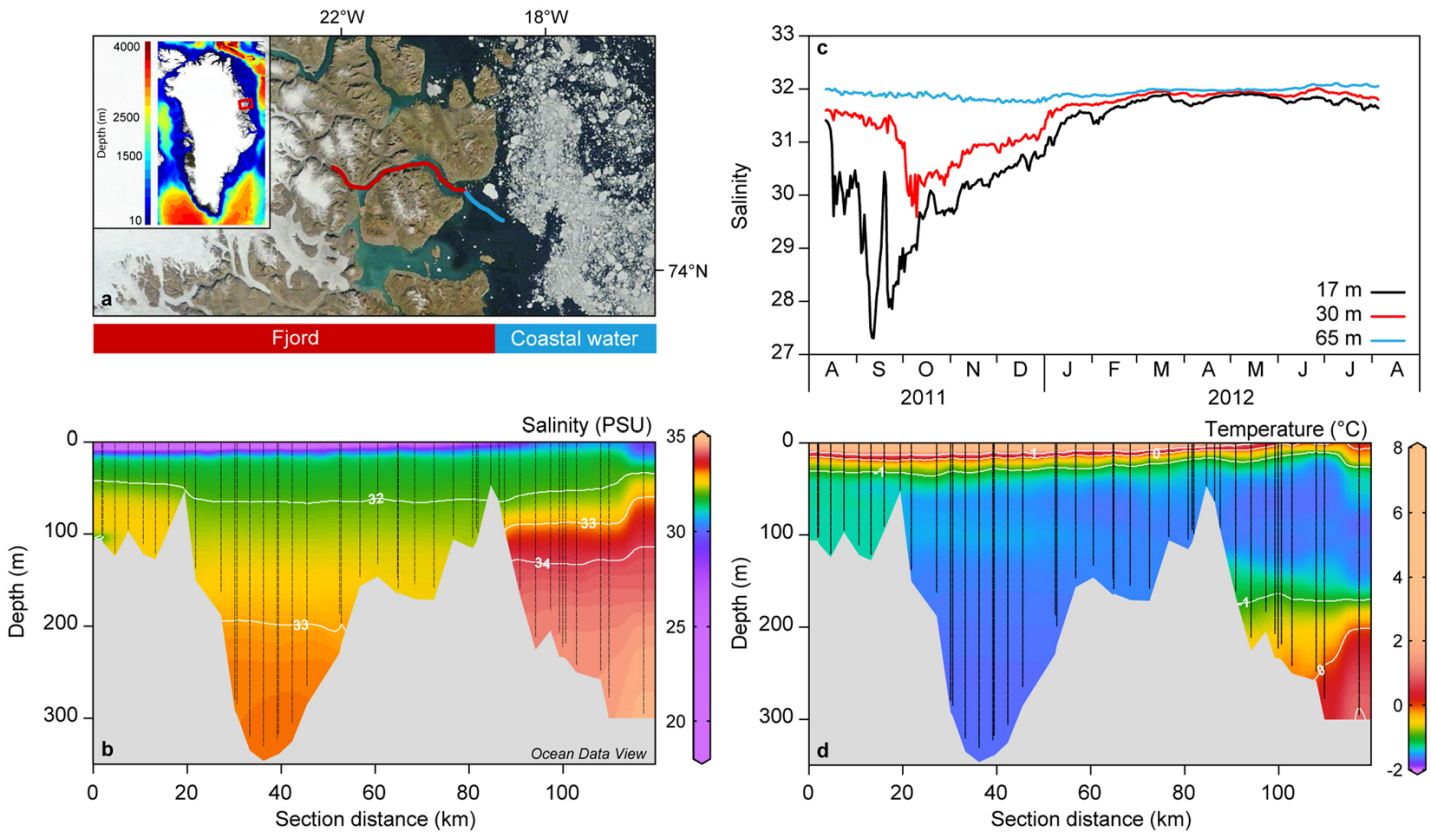
(**a**) Image of the study site in East Greenland with the hydrographical transect sampled from 2003 to 2015. (**b**) Locations of the sampling stations together with distribution in salinity for a typical year (2011). (**c**) Seasonal variability in salinity at three depths from a mooring in the fjord (near the 70 km mark in Fig. 1b) from 2011 to 2012. (**d**) Typical temperatures along the transect in August. Figure 1b and d where created using Ocean Data View version 4.6. http://odv.awi.de. Figure 1a satellite image credit: NASA Goddard Space flight Centre.

**Figure 2:**
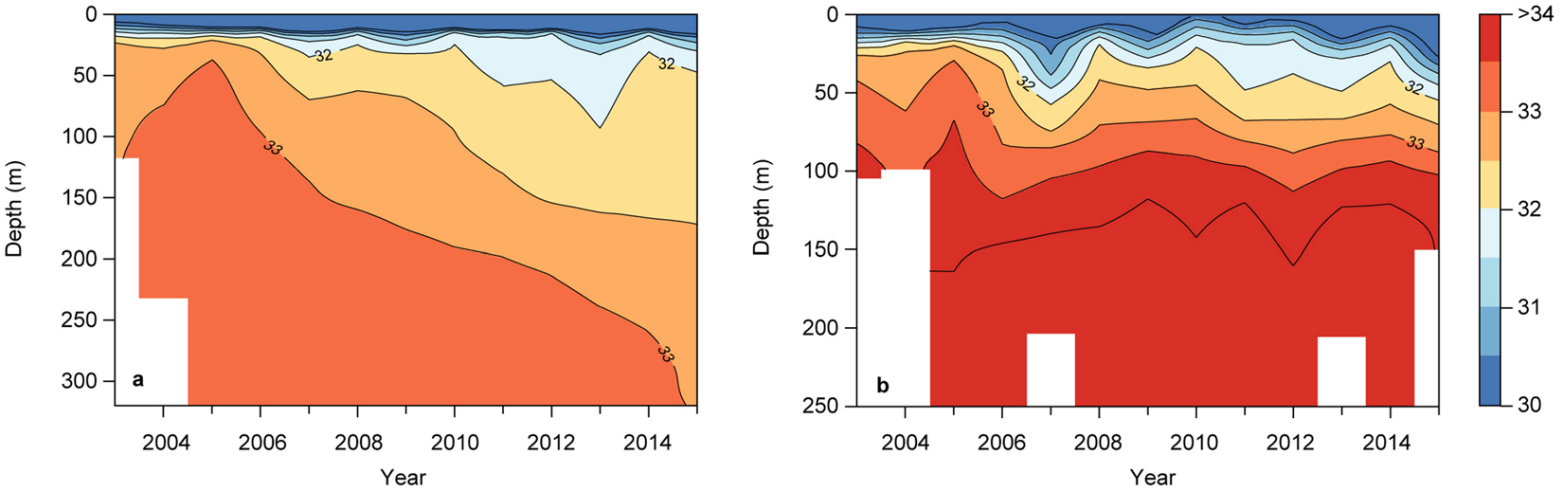
(**a**) Time-depth isopleths of average salinity over the study period for the fjord and (**b**) the coastal water of the hydrographical transect.

**Figure 3:**
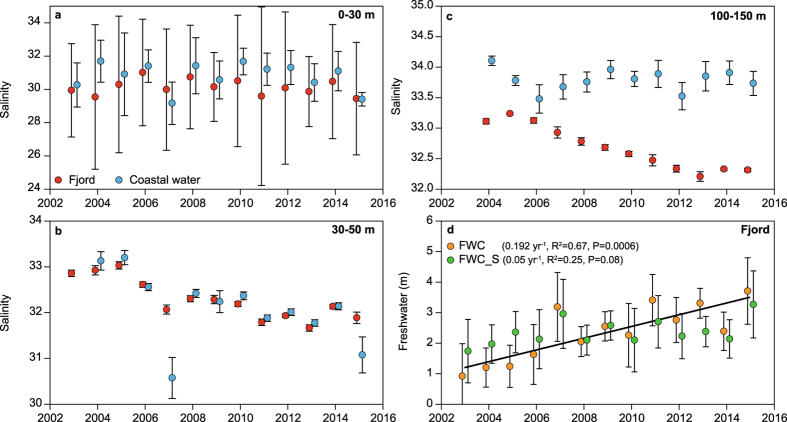
Changes in summer salinity of fjord and coastal waters at the study site in East Greenland for different depth strata: (**a**) 0–30 m; (**b**) 30–50 m and (**c**) 100–150 m. (**d**) Integrated estimate of freshwater content (FWC) in the top 50 m of the water column in the fjord. See text for definitions of FWC and FWC_S. The solid line represents the linear regression of FWC.

The Greenland Ecosystem Monitoring programme has conducted systematic hydrographic measurements in Young Sound (NE Greenland, 74.24° N 20.17° W, http://www.g-e-m.dk) since 2003. The aim of this study was to analyse the dataset for evidence in changes in the freshwater budget. The hydrographic transect is sampled each year in early August and spans from the inner fjord and approximately 30 km off the coast (Fig. 1a). The fjord system is divided into an inner shallow basin (Tyroler Fjord, to the west) and an outer deep basin (Young Sound) separated from the shelf by a shallow (45 m depth) sill (Fig. 1b). There are no marine terminating glaciers in the fjord, but several land terminating within the catchment. Freshwater from snow and glacial melt and precipitation is supplied through several rivers running into the fjord along its length supplying 0.9 to 1.4 km^3^ freshwater annually^9^. Coastal shelf waters in East Greenland are influenced by the East Greenland Current, which originates from the Arctic Ocean and follows the continental slope southwards. The current consists of Polar Surface Waters characterised by low salinities (S < 34.4) and sub-zero temperatures. Below the Polar Surface Waters, warm and saline re-circulating Atlantic Water is found typically at the depth of 250 m. For fjords such as Young Sound with a shallow sill, the dense Atlantic Water is prevented from entering the fjord as bottom water (Fig. 1d). In summer, the local freshwater contribution to the fjord is largely retained at the surface forming a shallow (5–10 m) surface lens with salinity below 25. The salinity and temperature of the surface waters are influenced by local atmospheric conditions, the presence of sea ice, melting snow and glacial meltwater discharge (Fig. 1b,d). We separated the transect into two sections: one inside the shallow outer sill (termed fjord) and one outside (coastal water). To visualize the inter-annual change we averaged all profiles for fjord and coastal water for each year to produce a contour plot of changes in average salinity profiles from 2003 to 2015. In the fjord, salinity decreased throughout the water column with a deepening of the 32 isohaline in the subsurface water and a deepening of the 33 isohaline near the bottom (Fig. 2a). In the coastal water (Fig. 2b), the most obvious change was in the subsurface water, where the 32 isohaline deepened after 2010 from approximately 25 to 50 m depth. Plots of average salinities in different depth strata, show no significant changes in surface waters (Fig. 3a), whereas the subsurface (Fig. 3b) and deeper layers (Fig. 3c) in the fjord show a gradual freshening by more than one salinity unit over the 13-year period. In the coastal water, the decrease is most pronounced in the 30–50 m layer (Fig. 3b). Linear regressions indicate a statistically significant decrease in salinity in the fjord of approximately 0.1 salinity unit per year at both 30–50 m and 100–150 m. Even in the bottom waters of the fjord (250–300 m) a significant decrease of 0.022 ± 0.005 per year is apparent (Table 1). Water temperatures in the different depth strata did not show a significant linear trend with the exception of the deepest part of the fjord, 250–300 m, where a significant warming was detected with a rate of 0.018 ± 0.001 °C yr^−1^. At 30–50 m in the coastal water, the significant linear decrease in salinity observed in the time series was 0.12 ± 0.05 per year. ﻿This is a significant freshening, of a magnitude similar to that found in the Beaufort Gyre^23^ To better assess the trend in freshening we calculated the integrated freshwater content (FWC) of the surface waters (0–50) in the fjord. We used the average salinity (2003 to 2015) at 50 m in Young Sound as the reference salinity. In the fjord, FWC for this layer increased from 0.9 ± 1.1 (2003) to 3.7 ± 1.1 m in 2015 (Fig. 3d).

**Table 1:** Result of linear regressions of changes in salinity and temperature 2003–2015 along the hydrographical transect (split into two; fjord and coastal waters) in NE Greenland. Significant regressions are shown in bold.

Depth	Fjord			Coastal waters		
	Alpha ± SE	R^2^	P	Alpha ± SE	R^2^	P
**Salinity**						
0–30 m	−0.044 ± 0.035	0.13	0.24	−0.033 ± 0.054	0.03	0.60
30–50 m	−0.099 ± 0.018	0.72	**<0.001**	−0.123 ± 0.048	0.35	**0.03**
100–150 m	−0.096 ± 0.009	0.92	**<0.001**	−0.008 ± 0.009	0.01	0.85
250–300 m	−0.022 ± 0.005	0.67	**<0.001**			
**Temperature**						
0–30 m	−0.106 ± 0.051	0.29	0.06	−0.012 ± 0.048	0.01	0.81
30–50 m	0.010 ± 0.009	0.11	0.27	0.004 ± 0.015	0.01	0.81
100–150 m	0.007 ± 0.007	0.10	0.32	0.018 ± 0.014	0.13	0.25
250–300 m	0.018 ± 0.001	0.99	**<0.001**			

**Figure 4:**
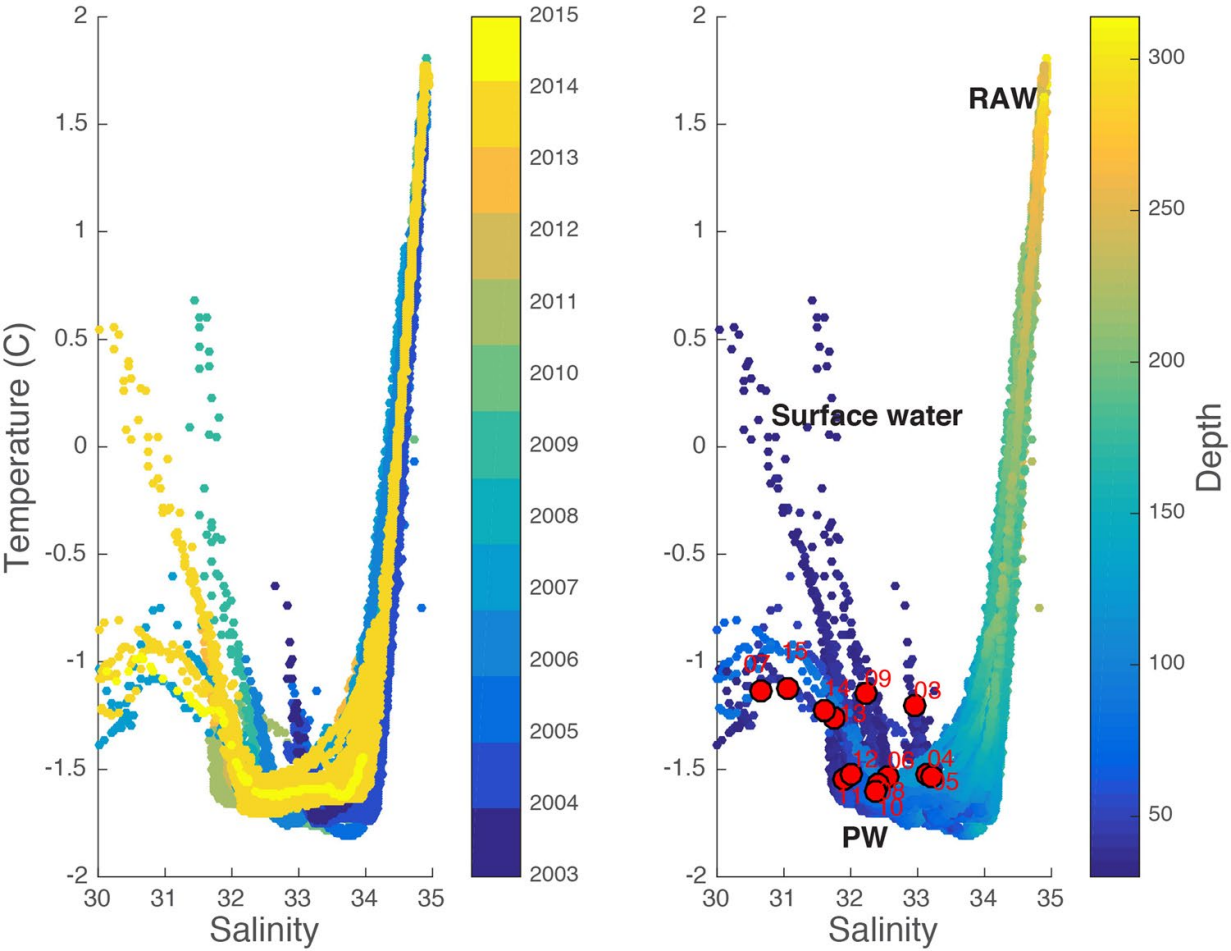
Temperature-Salinity plots of the subsurface (>30 m) coastal waters outside Young Sound in East Greenland. (**a**) The symbols are coloured by year and (**b**) by depth. PW: Polar water (T < 0, S < 34.4); RAW: Recirculating Atlantic Water (T > 0, S > 34.4). Superimposed on (**b**) are the average salinity and temperature values for coastal waters, at 30–50 m. (red dots, labelled with year).

There are two potential sources for the observed freshening in the fjord: from the local terrestrial catchment or the coastal water outside the sill. Data on total discharge from the main river in the catchment (Zackenberg River) reveals no significant increasing linear trend during the study period (Table 2, n = 13, p = 0.30).On average, more than 80 % of the annual discharge of the river has occurred by the date of the CTD survey with no evidence that the discharge up until that date has increased over the study period (Table 2, n=13, p=0.26). Maximum snow depth on land has not increased (n = 13, p = 0.83) and neither has the number of positive degree days (n = 13, p = 0.50). This indicates that snow and glacial melt of the catchment area in general is not increasing the runoff to the fjord. Wind driven vertical mixing can influence the vertical distribution of the freshwater. Young Sound typically becomes ice free just 3-4 weeks prior to the CTD survey and a strong halocline persists until autumn. It is therefore unlikely that inter-annual variation in vertical mixing before sampling is important for the observed decrease in salinity below 30m observed in August. We estimated the annual wind stress by integrating the daily average wind speeds during the ice free season (estimated from daily photos in the outer fjord) (Table 2). Neither this accumulated index of wind stress nor the duration of the ice free season revealed an increase for the period. Brine production (associated with a winter polynya outside the fjord) are an additional processes that may impact the seasonal and inter-annual variation in salinity along the studied transect﻿^24^ but further studies are required before their importance for the inter-annual patterns can be assessed. To further explore the source of freshwater we calculated the seasonal freshwater content, FWC_S, based on the average salinity at 50 m depth in Young Sound for each individual year (rather than the 2003–2015 average). In August, the salinity at 65  m is approximately equal to early spring conditions^7^ (Fig. 1c), and since the runoff is predominantly found in the upper 3﻿﻿0 m, and its residence time is relatively short^7^, the seasonal freshwater content becomes an estimate of runoff from each year. Thus, the differences between the FWC and FWC_S allow us to isolate a freshening driven from the fjord’s catchment, as FWC_S only represents freshening occurring from the winter until the time of sampling, driven by the local run off. As there is no significant change in FWC_S over the time series this calculation indicates that the freshening of the incoming water is driving the observed trends in FWC (Fig 3d). This supports the findings of no significant change in local river discharge, snow depth and degree days. Although there is considerable inter-annual variability in runoff from the catchment there is no clear indication of a significant increase in runoff to the fjord. The observed increase in freshwater content (FWC) of the fjord can, therefore, be explained by the observed freshening of the water masses at sill depth outside the fjord (30–50 m). This is also the depth range with the largest rate of decrease in salinity (Table 1). Plotted in temperature-salinity space this can be clearly seen as a migration of source waters to lower salinities with time (Fig. 4). The question remains as to the source of the freshening in the coastal water. The East Greenland Current is characterised by a core of Polar water with its origins in the Arctic Halocline and Polar Surface waters^10^. The freshwater present in this water mass originates from a combination of runoff from land, sea ice melt, precipitation, meltwater from the Greenland Ice sheet and freshwater input from the Arctic Ocean through the Fram Strait. In 2005, an increased transport of freshwater was observed in the Fram Strait (compared to 1998) which was attributed to the release of river water temporarily stored on the Siberian shelf^11^. In 2011 to 2013 an increasing contribution from Pacific water to the freshwater content in the Denmark Strait was observed^12^. Both these studies sampled on the East Greenland shelf whereas this study is close to the coast line in what has been termed the Riverine Coastal Domain in the Arctic Ocean^13^ characterized by coastal trapped runoff from land. The observed freshening along our transect, therefore, likely represents a regional signal of increased ice mass loss from the Greenland Ice sheet north of our study area which is accelerating due to increasing air temperature^5^. Clearly the meltwater signal from the ice sheet is modified by other fresh water sources. For example, the 2007 peak in FWC and low salinities coincides with an exceptional amount of melting sea ice in outer Young Sound (monitored by daily images in Young Sound) and also coincided with higher than average sea ice cover along the east Greenland coast^5^.

**Table 2:** Environmental data from the catchment area of Young Sound provided by the Greenland Ecosystem Monitoring Program.

Year	Degree days	Discharge 10^6^ m^3^	Discharge %	Snow depth m	Date of CTD	Ice free days	Wind stress m s^−1^
2003	406	189	85	0.6	Aug 11	128	379
2004	347	212	78	0.7	Aug 7	116	364
2005	117	167	89	0.7	Aug 8	88	287
2006	314	172	84	1.1	Aug 8	79	239
2007	339	183	84	0.6	Aug 10	74	205
2008	464	201	68	1.3	Aug 8	112	435
2009	374	146	78	0.2	Aug 3	90	309
2010	328	173	88	0.7	Aug 12	99	287
2011	304	197	81	0.4	Aug 6	101	330
2012	368	231	74	1.3	Aug 3	87	253
2013	405	147	78	0.1	Aug 12	105	456
2014	301	219	80	0.9	Aug 3	91	353
2015	398	268	89	1.1	Aug 16	92	307

Positive degree days are calculated by summing average daily temperatures (>0) each year until the date of the hydrographic survey in the fjord. River discharge is the accumulated annual discharge by the Zackenberg River running into the fjord system. Discharge, % is the proportion of the annual discharge that takes place up until the date of the CTD sampling. Snow depth, is the maximum snow depth measured in the catchment area. Date of CTD gives the date of the sampling of hydrographic properties in the fjord. Ice free days, gives the duration of the sea ice free season in the outer part of Young Sound, corresponding to the 60–80 km part of the section in Fig 1b. Wind stress was estimated by summing daily average wind speeds during the ice free season.

Meltwater from the Greenland Ice Sheet impacts the local marine ecosystem in numerous ways, providing organic carbon^14^ and nutrients^15^, influencing light availability^16^, productivity^17^ and pelagic^18^ and benthic^19^ ecosystem structure. Impacts of changing freshwater budgets in coastal waters is an additional manifestation of a warming climate, and in concert with changes in sea ice cover, ocean warming and acidification, freshening is likely an important driver for ecosystem change that remains poorly quantified in the Arctic.

The salinity time series presented are unique for the region and the first to document a decreasing trend in salinity on decadal scale from the coastal ocean surrounding the ice sheet. The presence of a freshening signal in Northeast Greenland despite a modest increase in ice sheet melt (17%, compared to 48% for southern Greenland^4^) suggest that meltwater is efficiently retained near the coast as has been shown in other Arctic regions^13^. Recent modelling studies have shown that meltwater from East Greenland may be efficiently transported to the Labrador Sea^20–22^. Combined with the decreasing salinity trend in our data it suggests that a signal of ocean freshening is being transported downstream by a nearshore component of the East Greenland current and will contribute to the freshwater content in the subarctic Atlantic.

### Methods

Hydrography profiles were obtained using a Sea-Bird SBE19plus CTD. The instruments were calibrated by Sea-Bird every year before fieldwork and data was averaged for 1 m intervals. For this analysis a database of about 300 CTD profiles has been accumulated and analysed. Salinity profiles in this analysis covers measurements during early August. The profiles were divided into two sectors; inside the fjord and outside in the coastal water masses. Only profiles with water depth larger than 50 m were considered. Freshwater content (FWC) was calculated as:

1 $${\rm{FWC}}=50\,{\rm{m}}\times (1-{S}_{m}/{S}_{{\rm{ref}}})$$

where S_ref_ was a reference salinity characterizing water in the surface layer before spring. From observation and model simulations it was shown that salinity in Young Sound was relatively constant in the upper 50 m during winter and variations below 30 m were relatively small during the year^9^. Therefore, the reference salinity was defined from the average value of S_m_ in the fjord at 50 m depth in the period 2003–2015 (S_ref_ = 32.36). An additional measure of the annual change of freshwater content (FWC_S) was defined by applying the annual averaged value of S_ref_ in Eq. 1 (S_ref_ = 32.91, 32.88, 33.12, 32.70, 32.20, 32.39, 32.38, 32.25, 31.87, 32.00, 31.73, 32.19, 32.05 in 2003–2015). Wind speed was collected by the ClimateBasis component of Greenland Ecosystem Monitoring program (GEM). Average wind speed (10-min intervals) collected at a height of 7.5 m available at the GEM database were used. The dates for sea ice melt and sea ice formations was also extracted from the GEM database, where an automatic camera system provides information on daily local ice conditions in the outer part of Young Sound.

### Data availability statement

The datasets generated during and/or analysed during the current study are available from the corresponding author on reasonable request.
